# A Comprehensive Assessment of the Biocompatibility and Safety of Diamond Nanoparticles on Reconstructed Human Epidermis

**DOI:** 10.3390/ma16165600

**Published:** 2023-08-12

**Authors:** Wiktoria Fraczek, Kacper Kregielewski, Mateusz Wierzbicki, Patryk Krzeminski, Katarzyna Zawadzka, Jaroslaw Szczepaniak, Marta Grodzik

**Affiliations:** 1 Department of Nanobiotechnology, Institute of Biology, Warsaw University of Life Sciences (WULS-SGGW), 02-787 Warsaw, Poland; wiktoria_fraczek@sggw.edu.pl (W.F.); mateusz_wierzbicki@sggw.edu.pl (M.W.); patryk_krzeminski@sggw.edu.pl (P.K.); katarzyna_zawadzka@sggw.edu.pl (K.Z.); 2 Faculty of Biology and Biotechnology, Warsaw University of Life Sciences (WULS-SGGW), 02-787 Warsaw, Poland; s200026@sggw.edu.pl; 3 Department of Pathology and Veterinary Diagnostics, Institute of Veterinary Medicine, Warsaw University of Life Sciences (WULS-SGGW), 02-787 Warsaw, Poland; jaroslaw_szczepaniak@sggw.edu.pl

**Keywords:** nanodiamond, EpiDerm^TM^, OECD, TG439

## Abstract

Diamond nanoparticles, also known as nanodiamonds (NDs), exhibit remarkable, awe-inspiring properties that make them suitable for various applications in the field of skin care products. However, a comprehensive assessment of their compatibility with human skin, according to the irritation criteria established by the Organization for Economic Cooperation and Development (OECD), has not yet been conducted. The purpose of this study was to evaluate if diamond nanoparticles at a concentration of 25 μg/mL, incubated with reconstituted human epidermis (EpiDerm^TM^) for 18 h, conform to the OECD TG439 standard used to classify chemical irritants. For this purpose, a cell viability test (MTT assay), histological assessment, and analysis of pro-inflammatory cytokine expression were performed. The results indicated that NDs had no toxic effect at the tested concentration. They also did not adversely affect tissue structure and did not lead to a simultaneous increase in protein and mRNA expression of the analyzed cytokines. These results confirm the safety and biocompatibility of NDs for application in skincare products, thereby creating a wide range of possibilities to exert an impact on the advancement of contemporary cosmetology in the future.

## 1. Introduction

In recent years, diamond nanoparticles, similar to some other carbon nanomaterials have gained increasing interest in biomedical and cosmetology fields due to their biocompatibility, high stability, and unique optical and chemical properties. These characteristics make them promising candidates for various applications [1,2,3]. NDs show potential in improving drug delivery, durability, and dispersion of insoluble therapeutic substances in water. They can also be used as additives in skincare formulations such as creams and balms. NDs facilitate the deep penetration of active ingredients into the skin, thereby enhancing their therapeutic effectiveness [4,5]. The unique optical properties of NDs also make them suitable for protection against ultraviolet (UV) radiation. They can reflect and scatter harmful UV radiation, making them potential candidates for use in sunscreen filters aimed at preventing certain types of skin cancer [6,7].

However, the chemical structure of the functional groups attached to the surface of NDs determines their properties, further increasing the complexity of their potential applications. An example of this is the hydrophilic properties of diamond powder, resulting from the presence of OH groups on the surface. These groups allow nanodiamond particles to bind to the skin surface, reducing water loss through the epidermis and positively influencing the function of the hydrolipidic layer and overall skin condition [8].

Additionally, diamond nanoparticles can enhance the longevity of cosmetics by delaying oxidation of unsaturated fatty acids and the lipid phase of cosmetics. Hydroxylated diamond nanoparticles protect cosmetics from the adverse effects of external factors such as light, oxygen, or elevated temperatures [9]. Despite the numerous potential applications of diamond nanoparticles, it is important to remember that evaluation of the toxicological effects of chemicals on the skin is a key requirement for their approval in commercial applications. This is especially important for compounds that have so far been tested mainly on 2D cellular and animal models [5,10].

One of the potential adverse effects that needs to be evaluated is skin irritation. According to the Globally Harmonized System of Classification and Labelling of Chemicals (GHS), this term refers to reversible damage to the skin that occurs up to several hours after the application of a substance [10,11]. Clinical signs of skin irritation include redness and swelling, which usually subside within a few days [12]. Traditionally, animal models such as the Draize test had been used to assess skin irritation [13]. However, the validity of these results in relation to humans has been questioned due to ethical concerns and reducing animal usage, leading to proposals for alternative methods [14]. In response to demand, the Organization for Economic Cooperation and Development (OECD) has developed guidelines for the validation of new methods, including the assessment of skin irritation [10].

An alternative test is to use in vitro models to evaluate possible skin irritation caused by chemicals, cosmetics, and other compounds. The EpiDerm^TM^ model is an advanced model of reconstructed human epidermis that uses unprocessed human-derived keratinocytes. This model accurately replicates the histology, cytoarchitecture, biochemical, and physiological properties of the upper layers of human skin, making it a valuable tool for evaluating the irritant properties of various substances without the need for animal testing [10,14,15]. The availability of standardized protocols allows for the validation and reproducibility of these methods, enabling the assessment of multiple parameters such as viability, barrier function, and tissue morphology [10].

The penetration of chemical substances through different layers of the skin, especially the epidermis and dermis, can lead to the release of pro-inflammatory cytokines. Keratinocytes, which constitute 95% of epidermal cells, are the main cells responsible for cytokine secretion upon skin stimulation, thus play a crucial role in the induction and development of irritant contact dermatitis [16,17]. It should be noted that further research and validation are needed to fully understand the interactions between nanodiamonds and human skin.

The purpose of the study was to comprehensively assess the biocompatibility and toxicity of diamond nanoparticles using a three-dimensional model of normal human epidermal tissue, which shows more similarity with human epidermis than 2D and animal models. The utilization of EpiDerm^TM^ in accordance with OECD guidelines during diamond nanoparticle research demonstrates a forward-looking approach that may enable broad and practical cosmetology applications of ND in the future. For this reason, special emphasis was placed on evaluating the tissue structure and potential pro-inflammatory effects according to the irritant test to determine the safety implications of ND applications on the skin.

## 2. Materials and Methods

### 2.1. Characterization of Diamond Nanoparticles

Detonation-produced diamond nanoparticles were purchased from SkySpring Nanomaterials (Houston, TX, USA). They had a purity of greater than 95% and a surface area of about 282 m^2^/g, according to the manufacturer’s specifications. A stock solution was prepared by suspending the nanodiamond powder in ultrapure water.

The shape and size of the diamond nanoparticles were examined using a JEM-1220 transmission electron microscope (TEM, JEOL, Tokyo, Japan) at 80 kV with a Morada 11-megapixel TEM CCD camera (Olympus Inc., Tokyo, Japan). Prior to imaging, a sample of 7 μL of 10 μg/mL ND was deposited on formvar-coated copper grids (Agar Scientific Ltd., Stansted, UK). The hydrocolloid was then allowed to air-dry.

X-ray photoelectron spectroscopy (XPS) was conducted using the PREVAC ultra-high vacuum system (Rogów, Poland) to analyze the elemental composition, functional groups, and chemical states on the surface of diamond nanoparticles. The spectra were obtained using a Scienta R4000 electron analyzer. Additional equipment was used, such as the VG Scienta SAX-100 X-ray source (Al Kα, 1486.7). Survey spectra were acquired with a pass energy of 200 eV (with 500 meV steps), while high-resolution spectra for specific regions (C1s, O1s, N1s) were obtained with a pass energy of 50 eV (with 50–75 meV steps). The base pressure in the analysis chamber was maintained below 1 × 10^−8^ mbar throughout the data collection process.

### 2.2. EpiDerm^TM^ and ND Exposure

EpiDerm™ (EPI-200), the reconstructed human epidermal tissues, were purchased from MatTek Corporation (Ashland, OR, USA). In this 3D tissue model, a semi-permeable tissue culture insert is used to cultivate normal human-derived epidermal keratinocytes (NHEK) at the air–liquid interface. The NHEK cells create a multilayered, highly differentiated replica of the human epidermis. Briefly, EpiDerm^TM^ tissues were placed in 6-well plates (Corning Inc., Corning, NY, USA) with 0.9 mL of maintenance medium, supplied by manufacturers with tissues. Prior to testing chemicals, the tissues were adjusted overnight at 37 °C, 5% CO_2_, in a humidified incubator. For the OECD TG439 [10] in vitro irritation test, the model samples were divided into 3 groups treated with 25 µL tested substances as follows: negative control (NC) treated with Dulbecco’s phosphate buffered saline (DPBS), positive control (PC) treated with 1% sodium dodecyl sulphate (Sigma-Aldrich, St. Louis, MO, USA), and experimental group treated with 25 µg/mL diamond nanoparticles (SkySpring Nanomaterials, Houston, TX, USA). The tissues were then incubated for 18 h as previously described by Bengalli et al. [18]. After the treatments, the tissues were removed from the incubator, rinsed thoroughly, and gently dried on blotting paper to remove any residual fluid that may still have contained the test substances. Tissues were stored at −80 °C for protein and gene analysis; however, for the viability test and histological examination, the tissues were used immediately.

### 2.3. Tissues Viability Assay

To examine tissue viability, the MTT assay was performed as recommended by the OECD TG439 in vitro irritation test [10]. For this purpose, 1 mg/mL stock solution of 3-(4,5-dimethylthiazol-2-yl)-2,5-diphenyltetrazolium bromide (MTT, Sigma Aldrich, St Louis, MO, USA) was prepared in appropriate medium provided with EpiDerm™. Immediately after washing each tissue in DPBS, the inserts were transferred to a 24-well plate containing 300 μL of a 0.3 mg/mL of MTT solution per well and placed in the humidified incubator with 37 °C, 5% CO_2_ for 3 h. Next, the inserts were blotted with absorbent paper and insoluble formazan products of MTT were extracted from the tissue by 2 mL isopropanol (Sigma Aldrich, St Louis, MO, USA) that was added to each well a new 24-well plate. After 3 h of gentle shaking on a plate shaker, 200 μL of the isopropanol extract was transferred to a 96-well plate, with eight repetitions per group. Using an Infnite^®^ 200 PRO microplate reader and i-control^TM^ 2.0 software (Tecan Group Ltd., Männedorf, Switzerland), color intensity was measured at 570 nm. By determining the percentage of tissue viability compared to the mean of the negative control, the irritating potential of ND was assessed. According to the Global Harmonised System (GHS) categorization [11], a chemical is considered an irritant of the second category if the obtained values are less than or equal to 50%; otherwise, they are considered non-irritants.

### 2.4. Tissues Viability Assay

The EpiDerm™ tissues were pre-fixed overnight at 4 °C in 10% paraformaldehyde. Alcohol gradient dehydration for histological characterization was performed by sequential treatment of 3 samples per group with 30% EtOH for 2 h, 50% EtOH for 2 h, 70% EtOH overnight, 95% EtOH twice for 3 h, and 100% EtOH twice for 1 h each time. Tissue samples were then sectioned and stained using standard HE staining.

The slides were examined and recorded using 20× and 40× magnifications with a Leica DM750 microscope, a Leica ICC50 digital camera, and cellSens microscope imaging software (Olympus Corporation, Warsaw, Poland).

The thickness of the corneal layer and the total EpiDerm™ thickness were defined as indicators of the appropriate activity and function of the model after treatment [19]. All measurements were performed in randomly selected fields of view at 20× magnification (12 photos per group). For histological examination, 40× magnification was used.

### 2.5. Inflammatory Cytokines Array

The impact of diamond nanoparticles on the pro-inflammatory cytokine response was assessed in EpiDermTM, Human Inflammation Antibody Array—Membrane (40 Targets) (ab134003) in triplicates. This test is based on antibody paired assay and gives results in duplicate for each membrane.

Before the procedure, frozen tissues were homogenized using a cell extraction buffer (Thermo Fisher Scientific, Waltham, MA, USA) and a Polytron^®^ PT 2100 homogenizer (Kinematica AG, Lucerne, Switzerland). The samples were incubated on ice for 30 min, with occasional vortexing, and then centrifuged for 10 min (4 °C, 13,000× *g* rpm). The total protein concentration was examined using the Modified Lowry Protein Assay (Thermo Fisher Scientific, Waltham, MA, USA) according to the manufacturer’s instructions.

The array membranes were washed in blocking buffer for 30 min and then incubated for 2 h with 200 µg of total proteins diluted in 1 mL of blocking buffer per membrane. Multiple washes were carried out according to the producer’s recommendations (Abcam ab134003, Cambridge, MA, USA). Subsequently, biotin-conjugated anti-cytokine antibodies were added and incubated for the next 2 h. After the washing steps, the membranes were incubated with HRP-conjugated streptavidin for 2 h. The entire procedure was carried out at room temperature. Chemiluminescence detection was used to visualize the results. Signals were detected with the Azure C400 system (Azure Biosystems, Dublin, Ireland).

The Protein Array Analyser programme for ImageJ software (Research Services Branch, National Institute of Mental Health, Bethesda, MD, USA) was used to densitometrically analyze the signals [20]. The computations were carried out in accordance with the manufacturer’s instructions to normalize the array data.

### 2.6. cDNA Synthesis and qPCR Analyses

For the isolation of total RNA, tissues were homogenized using a Polytron^®^ PT 2100 homogenizer (Kinematica AG, Lucerne, Switzerland) and PureLinkTM RNA Mini Kit (Thermo Fisher Scientific, Wilmington, DE, USA), according to the manufacturer’s protocol. The High Capacity cDNA Reverse Transcription Kit (Thermo Fisher Scientific, Waltham, MA, USA) was used to synthesize cDNAs from the extracted RNA under the following cycle conditions: 10 min at 25 °C, 120 min at 37 °C, and 10 min at 4 °C. Reverse transcription reaction was performed using a 2720 Thermal Cycler (Thermo Fisher Scientific, Waltham, MA, USA). The purity and concentration of cDNA were measured by the NanoDrop OneC spectrophotometer (Thermo Scientific, Wilmington, DE, USA).

The qPCR analyses were performed on a QuantStudio 5 thermal cycler (Thermo Fisher Scientific, Waltham, MA, USA) using Fast Probe qPCR Master Mix (Eurx, Gdańsk, Poland) with SG and ROX addition (Eurx, Gdańsk, Poland). The analyzed genes were selected based on a previously conducted protein array assay. Genomed (Warsaw, Poland) provided the gene-specific primers (Table 1) and *RPL13* was used as the reference gene. The amplification was carried out as follows: activation for 2 min at 50 °C, presoak for 10 min at 95 °C, followed by 40 cycles of a two-step PCR consisting of a denaturing phase at 95 °C for 15 s, and a combined annealing with extension phase at 60 °C for 1 min. The program implemented for the TNFα gene served as an exception, and its presentation was as follows: activation for 2 min at 50 °C, presoak for 8 min at 95 °C, followed by 40 cycles of a two-step PCR consisting of a denaturing phase at 95 °C for 15 s, annealing at 63 °C for 1 min, with an extension phase at 72 °C for 30 s. The relative gene expression (RQ) was computed using the formula 2^−ΔΔCT^.

### 2.7. Statistical Analysis

The data were analyzed by monofactorial analysis of variance (one-way ANOVA) for cytotoxicity and morphological analysis. Differences between the control and treated group were evaluated using Tukey’s HSD post hoc test. The Student’s *t*-test was utilized to analyze the expression of pro-inflammatory cytokines. The *p*-values ≤ 0.05 were considered significant. All the statistical analyses were performed using GraphPad Prism 9 (GraphPad Software Inc., La Jolla, CA, USA).

## 3. Results

### 3.1. Physicochemical Properties of Diamond Nanoparticles

**Figure 1:**
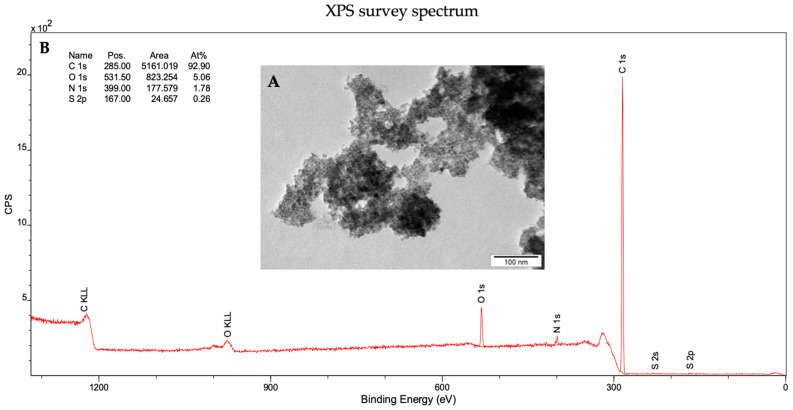
Physicochemical analyses of nanodiamonds. (**A**) Transmission electron microscopy (TEM) images; (**B**) Survey X-ray photoelectron spectroscopy (XPS) spectra of ND.

The morphology of diamond nanoparticles was validated using transmission electron microscopy (TEM). The obtained results indicated that the tested material had a spherical shape and slight tendency to form agglomerates (Figure 1A).

X-ray photoelectron spectroscopy (XPS) analysis provided information about the surface composition of diamond nanoparticles (Figure 1B and Figures S1–S3). The results indicated that the main component on the surface was carbon, accounting for 92.9 atomic percentage. Oxygen, with a value of 5.1%, was also an equally significant element in determining the physicochemical properties of diamond nanoparticles. Additionally, small amounts of nitrogen and sulfur were detected (1.8% and 0.3% atomic percentage, respectively).

### 3.2. Biocompatibility of Diamond Nanoparticles

**Figure 2:**
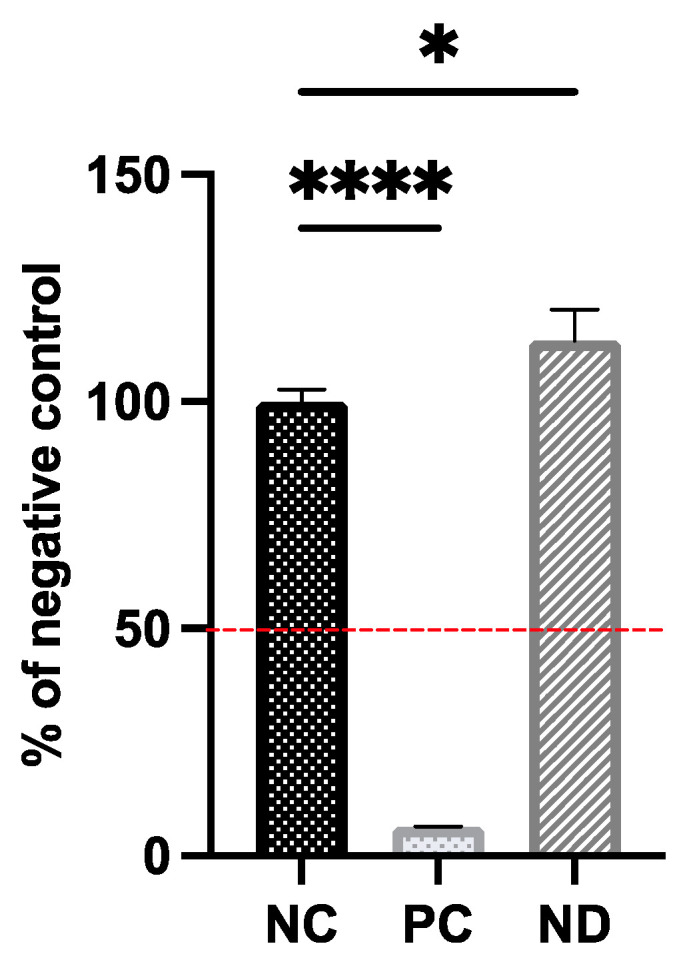
EpiDerm^TM^ viability evaluated by the MTT assay. The dashed red line indicates the critical point at 50% viability, indicating irritant potential. Mean ± standard deviation values are presented. Statistical significance between control and treated cells is indicated by an asterisk, evaluated using Tukey’s multiple comparison test (*p* < 0.05). Asterisk (*) represents *p* < 0.01, and four asterisks (****) represent *p* < 0.0001. Abbreviations: NC, negative control (DPBS treated); PC, positive control (1% SDS treated); ND, tissues treated with 25 μg/mL nanodiamond.

The viability of cells after exposure to the tested substance was evaluated by measuring the metabolic activity of mitochondria (Figure 2).

**Figure 3:**
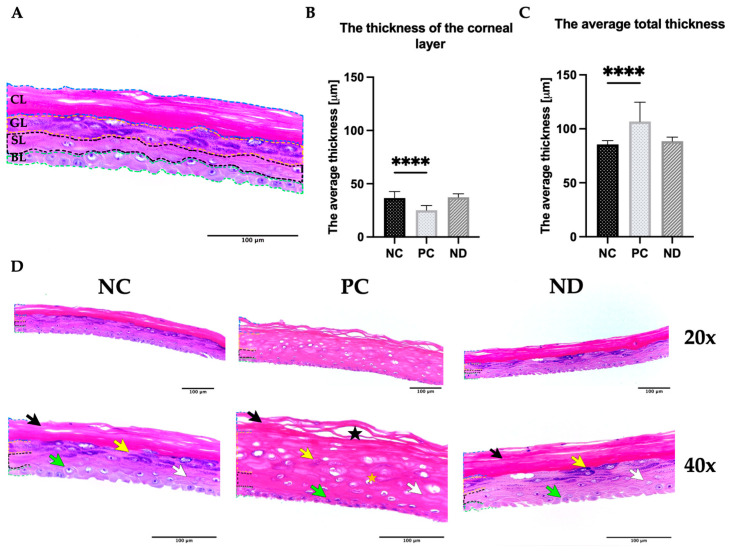
Histological examination of EpiDerm^TM^. (**A**) Scheme of tissue structure. With a dotted line, the individual layers are indicated as follows: blue—corneal layer (CL), yellow—granular layer (GL), black—spinous layer (SL), green—basal layer (BL); (**B**) average thickness of the stratum corneum layer; (**C**) overall thickness; (**D**) tissue visualization after hematoxylin-eosin staining at magnifications: 20× and 40×. The loosely arranged cells of stratum corneum are indicated as black arrows, lamellar bodies with keratohyalin granules are labeled as a yellow arrows, flattened cells of the spinous layer are pointed out with white arrows, the basal layer cells with a columnar shape with rounded contours are a green arrows. The tissue damage is indicated by a black star, and the orange star represents the presence of necrotic processes. Values are displayed as mean ± SD. Significant differences between control and treated cells are indicated with a *p*-value < 0.05, where **** denotes a *p*-value < 0.0001. Abbreviations: NC, negative control (DPBS treated); PC, positive control (1% SDS treated); ND, tissues exposed to nanodiamond at 25 μg/mL.

Based on OECD TG439, the results obtained after the MTT assay indicated that the hydrocolloid of diamond nanoparticles at a concentration of 25 μg/mL did not have an irritant potential against keratinocytes in the 3D model. Furthermore, the results indicated that there was a 10% increase in cellular metabolism of the test sample (ND) versus the negative control (NC). In contrast, tissues treated with 1% SDS (PC) showed negligible viability of approximately 6% compared to the negative control. Morphological analysis was also included as a step in assessing the biocompatibility of diamond nanoparticles. To examine the condition of all layers of EpiDerm™, tissue visualization was conducted following standard HE stains (Figure 3).

EpiDerm™ is a complex structure comprised of the stratum corneum, granular layer, spinous, and basal layers (Figure 3A). The visibility and proper staining of all these layers are evident in both the negative control tissues (treated with DPBS), and the EpiDerm™ exposed to diamond nanoparticles (Figure 3D). In addition, no inflammatory lesions were detected in any of these instances. The outermost layer of the skin, known as the stratum corneum, is characterized by observable layers of loosely attached cells. In the granular layer, the lamellar bodies appear normal, and the keratohyalin granules exhibit rounded to star-like shapes. Within the spinous layer, the cells are flattened, resembling the natural epidermis, while the cells in the basal layer have a columnar shape with rounded contours. However, representative images of SDS-treated samples (PC) showed multiple defects in the examined tissues. EpiDerm™ tissues treated with the irritant substance exhibited damage indicative of a necrotic process. Additionally, in some areas of the positive control tissue, a lack of compact and homogeneous morphology was observed in the stratum corneum layer. The evaluation of the total thickness of the examined samples, as well as the thickness of the stratum corneum layer, provided additional confirmation of the lack of significant irritant effect of nanodiamond on the structure of the used model. The evaluation of overall thickness and measurements of the stratum corneum revealed no discernible differences between the negative control samples and those treated with nanodiamond particles (Figure 3B,C). However, a markedly contrasting tissue response was observed following the application of SDS. In the negative control, the average values for these dimensions were 106 µm for the total thickness (21 µm higher compared to the negative control) and 25 µm for the width of the stratum corneum (11 µm lower compared to PC).

### 3.3. Evaluation of the Expression of Pro-Inflammatory Cytokines

**Figure 4:**
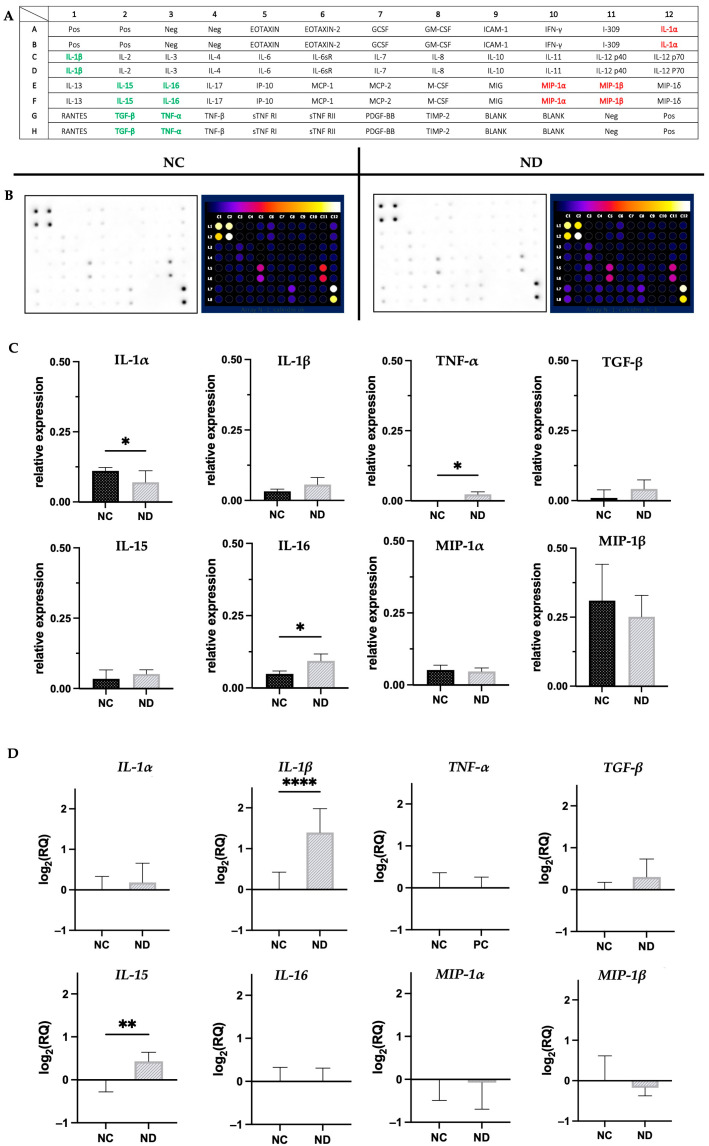
Proinflammatory cytokines expression. (**A**) Scheme of the protein pattern at the membranes. Proteins that showed an increase in expression after ND treatment are marked in green, whereas those exhibiting a decrease in expression are marked in red; (**B**) levels of cytokines after normalization; (**C**) the relative expression levels of selected proteins; (**D**) and their corresponding genes. Mean ± standard deviation values are presented. Statistically significant differences between control and treated cells are indicated by an asterisk, evaluated using Student’s *t*-test (*p* < 0.05). Asterisk (*) represents *p* < 0.01, (**) *p* < 0.05, while four asterisks (****) represent *p* < 0.0001. Abbreviations: NC, negative control (DPBS treated); ND, tissues exposed to nanodiamond at a concentration of 25 μg/mL; IL-1α, Interleukin-1 alpha; IL-1β, Interleukin-1 beta; TNF-α, Tumor necrosis factor; TGF-β, Transforming growth factor beta; IL-15, Interleukin-15; IL-16, Interleukin-16; MIP-1α, Macrophage inflammatory protein-1 alpha; MIP-1β, Macrophage inflammatory protein-1 beta.

The influence of the factors examined on the appearance of inflammation was investigated after an 18-h incubation of EpiDerm^TM^ tissues with 25 µg/mL ND. The study involved analysis of protein and mRNA levels. However, the SDS-treated samples showed a significant level of degradation that prevented extraction of the proper amount of protein and genetic material and led to their exclusion from the analyses. The use of protein arrays allowed for the screening of 40 cytokines (Figure 4A). Representative images (Figure 4B) selected from three repetitions indicated changes in the expression of the examined proteins under the influence of the applied factor.

Incubation with diamond nanoparticles was observed to induce the expression of tumor necrosis factor alpha (TNF-α), transforming growth factor (TGF-β), as well as several interleukins, including IL-1β, IL-15, and IL-16. A reduction of interleukin 1 alpha (IL-1α) and examined macrophage inflammatory proteins (MIP1α, MIP1β) compared to the control were observed (Figure 4C). Normalization of the results allowed for further quantification that revealed a statistically significant change in the proteomic levels of TNF-α, IL-1α, and IL-16. Additionally, both tissues treated with diamond nanoparticles and those in the control group demonstrated a notably high relative expression level of MIP1β.

The performed analyses allowed for the selection of cytokines that were further analyzed at the mRNA level of *IL-1α*, *IL-1β*, *TNF-α*, *TGF-β*, *IL-15*, *IL-16*, *MIP-1α*, and *MIP-1β* (Figure 4D). IL-15 and IL-1β were the only genes among the ones examined which demonstrated a 0.5- to 1.5-fold increase in expression following ND treatment.

## 4. Discussion

Nanoparticles originating from the same material can exhibit diverse physicochemical properties, as they depend not only on the constituent, but also on factors such as size, shape, tendency of aggregation, surface structure, and chemical composition [23]. To determine the properties of the investigated diamond nanoparticles, the focus was placed on visualization and qualitative analysis of the elements present in the surface layer. Detonation-produced nanodiamonds are known for their tendency to aggregate. It has been confirmed that aggregates ranging in size from 10 to even 500 nm consist of primary nanoscale particles with dimensions of 4–5 nm [4,24,25]. Additionally, depending on the method of synthesis and subsequent treatment, NDs can have various surface functional groups and outer coatings formed by disordered sp^2^ and sp^3^ carbon phases of different thicknesses [26]. A peak model for the high-resolution spectrum of the C1s region was based on the works of Koinuma and Xie [27,28]. In this region, the aliphatic C-C sp3 (C1sA) and C-H sp3 (C1sB) groups dominated, observed at 285 and 286.06 eV, respectively. Furthermore, the presence of aromatic carbon (C1sB, 284.46 eV) was identified, accounting for 22% of all visible carbon forms in the analyzed sample. Carboxyl groups (C1sD, 287.76 eV) and defected structures on the surface of the nanodiamond, removed due to the action of the solution (C1s deff., 283.47 eV), constituted a small atomic concentration of around 1.8%. The surface of the diamond nanoparticles was partially oxidized. The high-resolution spectrum of the O1s region revealed five distinct peaks that corresponded to quinones (O1sA; ~530.37 eV), carboxylic, carbonyl, carbonates, or hydroxides (O1sB; ~531.57 eV), epoxides, ethers, hydroxyl groups bound to aliphatic carbon (O1sC; ~532.49 eV), aromatic hydroxyl and carboxyl groups (O1sD; ~533.6 eV), and oxide anion (O1sD; ~529.01 eV) [29,30,31,32,33,34]. The carboxylic and carbonyl groups were the most abundant, representing nearly 40% of all oxygen forms present on the surface of the analyzed sample. Furthermore, the presence of amine (N1sA; 399.06 eV), pyridinic nitrogen (N1sB; 400 eV), and small amounts of N-oxide pyridine (N1sC; 402.9 eV) was detected on the surface of the diamond nanoparticles [35,36,37]. The findings align with the manufacturer’s specifications, which state the presence of diamond nanoparticles contain functional groups such as -OH, -COOH, -C-O-C, and -C=O on their surface. Detonation-produced diamond nanoparticles, purchased from SkySpring Nanomaterials, have found extensive applications in various fields. One potential application of these compounds lies in their use to investigate undesirable cytotoxicity in normal cells and assess their ability to induce expected cytotoxic effects while also modulating the expression of factors that improve invasiveness in diverse cancer cell lines [38,39,40,41]. Furthermore, safety studies conducted on rats demonstrated that the intraperitoneal administration of 500 μg/mL of these diamond nanoparticles did not have any toxic effects on blood parameters or rat growth, suggesting their potential use as remedies or in drug delivery systems [42]. However, it should be noted that their safety has not yet been confirmed in accordance with the OECD standard in epidermal application.

Our observations suggest that a concentration of 25 μg/mL of nanodiamonds does not exert a toxic effect on EpiDerm™ cells. However, the application of excessively high concentrations of nanoparticles in in vitro studies can have a negative impact on cellular respiration and metabolic activity. The primary parameter utilized in determining the optimal dose is the assessment of cytotoxicity [23]. Schrand et al. were among the first to examine the impact of detonation of diamond nanoparticles, sized between 2 and 10 nm, at 25, 50, and 100 μg/mL on cell cytotoxicity. Their study, employing the MTT assay, demonstrated the remarkable biocompatibility of these nanoparticles towards alveolar macrophages. Furthermore, it was shown that the viability of cells remained unchanged when exposed to 25 μg/mL of these nanoparticles [37]. The results of these studies were also confirmed in an analysis conducted by Mitura et al. During the evaluation of ND cytotoxicity, according to the ISO 10993-5 standard [43], they demonstrated that L929 fibroblasts exhibited high viability, normal morphology, and no changes in cell density after nanoparticle treatment [44]. Given that diamond nanoparticles can simultaneously exert a therapeutic effect on cancer cells, these findings are of significant interest [45]. However, in the analysis of substances that have potential applications for the skin, it is crucial to conduct studies on keratinocytes, the primary cell type found in the epidermis, as well as fibroblasts, the predominant cell type in the dermis [16,46]. Research conducted on immortalized human keratinocytes (HaCaT) demonstrated that the viability of cells treated with different concentrations of diamond nanoparticles remained unaffected [47].

Furthermore, the applied hydrocolloid may have a positive influence on the metabolic activity of keratinocytes in the tissue. Similar effects were observed by Mytych et al. who studied a normal diploid human facial skin fibroblast cell strain (FSF1). The study revealed that low concentrations of diamond nanoparticles (0.5 μg/mL) had a positive influence on cells, leading to an increased proliferation rate and metabolic activity [48]. These results are consistent with observations made using other carbon-based nanomaterials as well. Pulskamp et al. demonstrated a 20% increase in viability of rat (NR8383) alveolar macrophages after treatment with 5 mg/mL of single-walled carbon nanotubes, as indicated by the MTT assay [49].

To confirm the absence of negative impact of diamond nanoparticles on tissues, it was necessary to evaluate the structural integrity and barrier function of the epidermal barrier. Verification of these parameters can be achieved by histological examination [50]. The results obtained from the treatment of EpiDerm™ with diamond nanoparticles align with the results of the MTT assay and indicate no impact of the factor tested on tissue. No significant differences were observed between the negative control and the samples treated with nanodiamonds in terms of all parameters evaluated. The evaluated layers showed a consistent structure and the cellular morphology within them appeared to be intact. The results concerning normal tissues are consistent with the observations made by Zieliska et al. after incubation of EpiDerm™ with PBS [51]. Moreover, for both examined groups, the measurements of overall width were in agreement with the anticipated results, ranging from 83 to 100 μm [19].

The results obtained from in vivo studies support our findings, indicating that the histology of the skin remains unaffected following treatment with diamond nanoparticles. Analyses using optical coherence tomography demonstrated that mouse skin samples incubated with ND for 24 h maintained a proper structure and did not differ significantly from tissues in the control group. Additionally, these analyses suggest that diamond nanoparticles can traverse the skin barrier and penetrate through the layers of the epidermis [52]. Moreover, prolonged exposure to modified nanodiamonds exerts a protective effect on the skin of guinea pigs treated with nickel and cobalt ions. This reduces the probability of an allergic reaction, which often occurs as a result of irritation [16,53]. However, the comparison between human and mouse skin tissue highlights the presence of significant species differences, emphasizing the importance of considering these variations when utilizing mice as a substitute model for understanding human physiology [54]. Mitura et al. performed patch tests on human back skin to investigate the possible irritation and allergic reactions caused by diamond powder particles. Their findings provided evidence that diamond powder is a biocompatible biomaterial for human skin [55].

On the contrary, a distinct tissue response can be observed after the application of irritating substances. EpiDerm™ exposed to SDS (PC) exhibited characteristics indicative of progressive tissue damage, which corresponds to the results obtained from the MTT test. The outermost layers, stratum corneum and stratum granulosum, are most susceptible to potential epidermal damage [56]. Therefore, the observed thinning of the outer layer in PC is one of the initial responses of the tissue to applied chemicals, indicating loss of barrier function and increased susceptibility to loss of water [57]. Upon contact with highly irritating substances, the lower layers of the epidermis (stratum spinosum and basal layer) may also undergo degradation [58]. One of the early visible indications of their damage is cell or cytoplasmic swelling [59]. This suggests that the observed increase in overall tissue thickness following exposure to SDS may be indicative of developing spongiosis. Histologically, contact dermatitis resulting from irritation typically presents with mild spongiosis, ranging from microscopic foci to visible vesicles, as well as necrosis of the epidermal cells [60]. However, visible degradation is a typical reaction for tissues treated with an irritating substance, which serves as a positive control [51,61]. The absence of such changes in tissues treated with diamond nanoparticles indicates their safety for use on the skin.

To reliably confirm this hypothesis, it was essential to perform analyses based on the expression of anti- and pro-inflammatory cytokines, which are released early in the cellular response, when tissue damage may be barely visible or even undetectable. Therefore, cytokines are considered suitable markers for objective and early detection of inflammatory reactions resulting from irritation and other factors [62,63]. It is also noteworthy that keratinocytes, which are the main cells of the epidermis, play a crucial role in cytokine production [64]. TNF-α and IL-1 are recognized as the main cytokines associated with inflammation. They initiate a signaling cascade that induces gene expression, synthesis, and release of secondary mediators such as various cytokines, chemokines, or growth factors [65,66]. TNF-α, produced by various types of cells, including fibroblasts and keratinocytes, is a pro-inflammatory protein. It is induced in the epidermis after exposure to harmful stimuli such as UV radiation or chemicals [67,68]. Before release, the protein precursor of TNF-α transforms into an active form that binds to cell surface receptors, resulting in various metabolic changes. This cytokine is considered crucial in initiating inflammation and tissue degradation, thus plays a significant role in irritant reactions. Increased expression of TNF-α has been observed in response to allergens and irritating sensitizing factors [69,70]. Protein and mRNA analyses demonstrate minimal levels of TNF-α in the DPBS-treated negative control. The slight elevation in protein levels observed in the ND-treated group is likely a short-term response to the stimulus and the stability of the resulting structure. The active form of the peptide can persist at elevated levels for an extended period by binding to cell surface receptors, even in the absence of an ongoing inflammation, as confirmed by gene analyses [71,72]. Notably, there was no detection of TNF-α gene expression after an 18-h incubation of EpiDerm™ with diamond nanoparticles. In vivo studies conducted by Wu et al. also support the safety of diamond nanoparticles in dermal applications, as shown by ELISA, indicating no increase in TNF-α levels in mouse skin treated with ND [7]. Similarly, experiments on a macrophage cell line (RAW 264.7) exposed solely to diamond nanoparticles show no significant increase in TNF-α production [73].

The production of IL-1α, which is correlated with TNF-α, has been validated by Magcwebeba et al. using well-known anti-inflammatory drugs to create a screening model to identify skin irritants [66]. The production of IL-1α in keratinocytes is increased in response to various stimuli, including exposure to different chemical compounds and UVB radiation [66,74,75]. The lack of a noticeable increase in IL-1α expression and its corresponding gene indicates that nanoparticles do not possess irritant potential. Moreover, a statistically significant reduction in protein levels suggests that the hydrocolloid used in this context may act as a pan-cytokine sponge. Similar findings were reported by Yoo et al. in their research on sepsis patients’ serum samples [76].

Levels of IL-16 exhibited a similar trend to TNF-α. Our studies showed an increase in protein levels in response to diamond nanoparticles. However, at the mRNA level, there was no expression of this cytokine in either the treated or control groups. IL-16 is produced by skin cells, particularly keratinocytes, during allergic reactions, but our findings suggest that diamond nanoparticles do not affect the prolonged immune response that leads to sensitization [77]. It is worth noting that IL-16 has multiple functions, including its ability to promote pro-inflammatory cytokines such as IL-15 [78].

Early findings on the role of IL-15 in keratinocytes indicated that increased expression of this cytokine occurs in instances of inflammation or abnormalities caused by excessive exposure of the skin to UVB radiation [79]. However, over time, the perception of interleukin 15 as exclusively pro-inflammatory has shifted. IL-15 is consistently present in keratinocytes and is regarded a crucial growth factor and attractant for leukocytes [80,81]. Thus, this cytokine plays a significant role in both normal skin biology and pathological skin processes [82]. However, IL-15 remains inactive until it binds to a receptor on the cell membrane, which is why it may not be detectable in certain in vitro studies involving the analysis of cytokine release in culture medium [81]. Research suggests that human mesenchymal stem cells derived from adipose tissue did not show any IL-15 expression, regardless of whether they were in a control group or treated with diamond nanoparticles. However, these results could be influenced by the use of conditioned medium during the experiments [83]. In robust inflammatory reactions, IL-15 is released as a result of cell death, which explains the high levels of this cytokine in the serum of patients with pneumonia. Interestingly, when serum was incubated with NDs, the expression of IL-15 decreased significantly, indicating that highly purified NDs can act as a sponge for various cytokines in plasma [76]. Analysis of the proteins isolated from EpiDerm^TM^ homogenates showed a slight increase in IL-15 levels in the skin treated with ND nanoparticles compared to the negative control. However, a significant and notable increase in IL-15 gene expression was observed in tissues exposed to diamond nanoparticles, suggesting that IL-15 has a dual function. These findings are consistent with the results of the MTT assay, which demonstrated higher cell viability in the tested sample. IL-15 is known to be a potent inhibitor of apoptosis in different cell types, including epithelial cells [81]. In normal circumstances, keratinocytes undergo apoptosis during their migration towards the skin surface and subsequent shedding [84,85]. The increased cell viability observed in the tested sample could be attributed to the slight elevation of IL-15 expression, which potentially slows down these processes. Another important point is that IL-15 has the capacity to induce IL-1β [86]. This aligns with our findings, which demonstrate a significant elevation in the expression of the IL-1β gene in tissues treated with ND. Although this cytokine is recognized primarily as a factor that regulates immune and inflammatory responses, studies have shown that IL-1β can trigger apoptosis and autophagy through the mitochondrial pathway [87,88]. The incubation of cells with metal particles for 24 h led to an increased expression of interleukin 1β. These findings were associated with the presence of a significant number of late apoptotic cells [89]. Interestingly, Blabler et al. did not observe increased expression of this cytokine in mesenchymal stem cells treated with ND [83]. Similarly, Thomas et al., in their study on the RAW 264.7 cell line, observed a reduction in IL-1β expression following exposure to diamond nanoparticles [90]. This suggests that the outcome we observed is specific to EpiDerm^TM^ and represents the tissue’s response to heightened IL-15 expression. In normal physiological conditions, the epidermis undergoes shedding, such that the obtained result may be the tissue’s mechanism for regulating the mRNA level to ensure homeostasis, rather than being a result of irritation.

Furthermore, the analysis of three other pro-inflammatory proteins synthetized or secreted during inflammation supports this hypothesis [91,92]. The levels of TGFβ, MIP1α, and MIP1β remained relatively stable in both the negative control group and the samples treated with nanodiamonds. The genes encoding these proteins exhibited a similar pattern. TGF-β is naturally expressed in all skin cells and contributes to tissue homeostasis, playing a vital role in the regulation of the skin barrier [93,94,95]. It has been shown that during injury or inflammatory stimuli, TGF-β acts as a mediator, influencing reactions in adjacent tissue compartments. Consequently, it plays an important role in skin remodeling after damage [96]. Moreover, it is hypothesized that ND may interact with one of the TGF-β receptors, thereby blocking its signal transduction [97]. This could explain the steady expression of TGF-β in EpiDerm^TM^ in response to the application of nanodiamond hydrocolloid.

Chemokines also play an important role in various processes, including allergic reactions, inflammation, and infections [97]. MIP1α and MIP-1β are considered among the most important chemokines, belonging to the CC chemokine group characterized by two adjacent cysteine residues near the amino group. These chemokines, particularly macrophage inflammatory protein-1, stimulate the migration of immune cells such as neutrophils, monocytes, and macrophages to sites of tissue damage [98]. Research using primary human macrophages (HMDM) and a human monocyte cell line (THP-1) demonstrated that surface-modified gold nanoparticles trigger the production of MIP-1α while having minimal impact on MIP-1β [99]. Similarly, studies with carbon nanotubes revealed that endotracheal administration of carbon nanomaterials to mice results in a dose-dependent increase in MIP-1α expression [100]. Notably, recent reports have revealed the absence of detectable levels of both MIP1α and MIP-1β in conditioned media following prolonged exposure of mesenchymal stem cells derived from adipose tissue to diamond nanoparticles [83]. Our results suggest that the consistent expression of MIP1 is a natural characteristic of tissue physiology, and it is noteworthy that diamond nanoparticles do not contribute to an increase in the levels of any of the chemokines investigated.

## 5. Conclusions

Our results indicate that the presence of diamond nanoparticles at a concentration of 25 μg/mL does not have a negative impact on the viability and morphology of three-dimensional keratinocyte cultures that simulate human epidermis. The use of all techniques outlined in the OECD TG439 guideline supports the conclusion that these nanoparticles do not exhibit irritant properties in the EpiDerm™, confirming their safety for use on skin. Analysis of key cytokine markers of skin irritation such as TNF-α and IL-1α demonstrates the absence of sustained and pronounced pro-inflammatory or allergic responses. No significant increase in protein and mRNA expression were observed for any of the analyzed cytokines. Furthermore, our results suggest that regulation of apoptosis at the molecular level may occur in the studied samples. Under physiological conditions, this process leads to natural exfoliation, preventing excessive keratinization.

## Figures and Tables

**Table 1:** Sequence of primers used in the qPCR analysis.

Gene	Sequence	Source
*RPL13*	F: CATAGGAAGCTGGGAGCAAG R: GCCCTCCAATCAGTCTTCTG	[21]
*IL-1α*	F: TGTATGTGACTGCCCAAGATGAAG R: AGAGGAGGTTGGTCTCACTACC	sp
*IL-1β*	F: CCACAGACCTTCCAGGAGAATG R: GTGCAGTTCAGTGATCGTACAGG	sp
*TNF-α*	F: GGCTCCAGGCGGTGCTTGTTC R: AGAGGCGATGCGGCTGATG	[22]
*TGF-β*	F: TACCTGAACCCGTGTTGCTCTC R: GTTGCTGAGGTATCGCCAGGAA	sp
*IL-15*	F: AACAGAAGCCAACTGGGTGAATG R: CTCCAAGAGAAAGCACTTCATTGC	sp
*IL-16*	F: TTGGACACAGGGTTCTCGCTCA R: AGCAGGGAGATAACGGACTGAC	sp
*MIP-1α*	F: ACTTTGAGACGAGCAGCCAGTG R: TTTCTGGACCCACTCCTCACTG	sp
*MIP-1β*	F: GCTTCCTCGCAACTTTGTGGTAG R: GGTCATACACGTACTCCTGGAC	sp

F, forward, sequence 5′ -> 3′; R, reversed, sequence 3′ -> 5′; sp, self-projected; *RPL13*, Ribosomal protein L13a; *IL-1α*, Interleukin-1 alpha; *IL-1β*, Interleukin-1 beta; *TNF-α*, Tumor necrosis factor; *TGF-β*, Transforming growth factor beta; *IL-15*, Interleukin-15; *IL-16*, Interleukin-16; *MIP-1α*, Macrophage inflammatory protein-1 alpha; *MIP-1β*, Macrophage inflammatory protein-1 beta.

## Data Availability

The data presented in this study are available upon request to the corresponding author.

## Supplementary Materials

The following supporting information can be downloaded at: https://www.mdpi.com/article/10.3390/ma16165600/s1, Figure S1: O 1s XPS spectra of an ND; Figure S2: C 1s XPS spectra of an ND; Figure S3: N 1s XPS spectra of an ND.
