# Supplementary figures and images for: A Comprehensive Assessment of the Biocompatibility and Safety of Diamond Nanoparticles on Reconstructed Human Epidermis
Source: Materials (Basel). 2023 Aug 12;16(16):5600. doi: 10.3390/ma16165600 (PMC10456456; doi:10.3390/ma16165600)

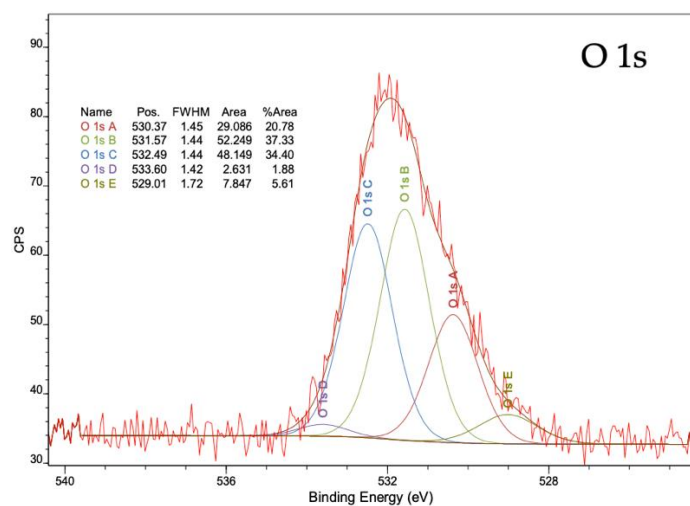

**Figure S1.** O 1s XPS spectra of an ND.

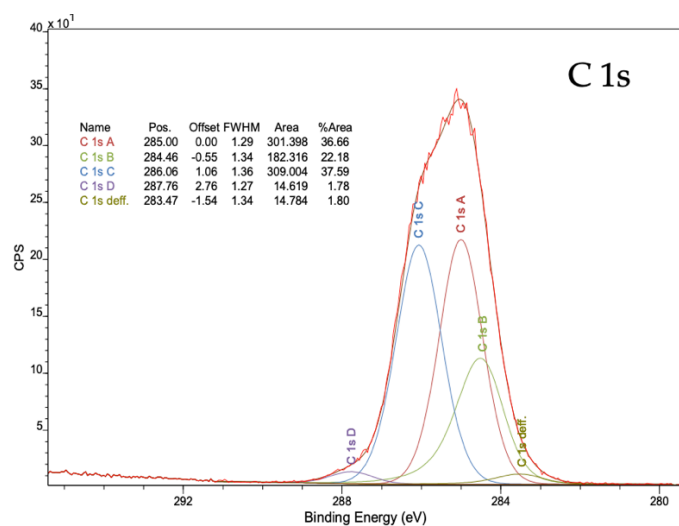

**Figure S2.** C 1s XPS spectra of an ND.

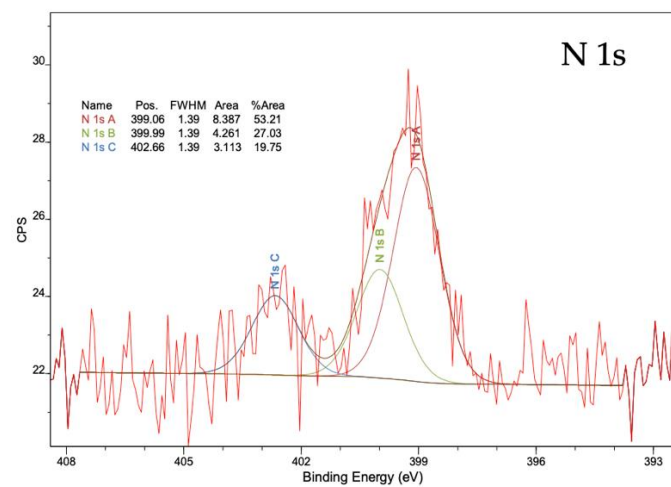

**Figure S3.** N 1s XPS spectra of an ND.

Supplement: Supplementary file 1 [file materials-16-05600-s001.zip › materials-2533382-supplementary.pdf]
